# Assessing the willingness of Nigerian dentists to adopt salivary diagnostic kits: a pilot cross-sectional study

**DOI:** 10.1186/s12903-026-07981-9

**Published:** 2026-02-27

**Authors:** Olubusayo Bolarinwa, Adetola Emmanuel Babalola, Olayinka Julianah Onasanya, Victor Miracle Johnson, Torojah Mayaline Williams, Oluwaseun Akinola Azeez, Victor Adeyanju Somoye, Abraham Oloture Ogwuche, Yakub Burhan Abdullahi, Kudirat Abike Giwa, Adaeze Favour Egemonye

**Affiliations:** 1 Johns Hopkins University, Maryland, USA; 2 Faculty of Dentistry, University of Ibadan, Ibadan, Nigeria; 3 Kornberg School of Dentistry, Temple University, Philadelphia, USA; 4 Institute for Health and Equity, Medical College of Wisconsin, Milwaukee, WI USA; 5 College of Medicine, University of Ilorin, Ilorin, Nigeria; 6 Faculty of Health Sciences and Tropical Medicine, Somali National University, Mogadishu, Somalia; 7 College of Medicine, University of Lagos, Lagos, Nigeria

**Keywords:** Ancillary diagnostic kits, Dentists, Nigeria, Oral health, Saliva diagnostics, Willingness to adopt

## Abstract

**Background:**

Saliva has emerged as a valuable diagnostic fluid because of its non-invasive collection, cost-effectiveness, and the presence of multiple biomarkers. Despite its clinical potential, the use of saliva-based diagnostics in low- and middle-income countries, including Nigeria, remains limited.

**Objective:**

This pilot study assessed Nigerian dentists’ awareness and willingness to adopt ancillary salivary diagnostic kits in dental practice.

**Methods:**

A descriptive cross-sectional survey was conducted among 119 licensed dentists in Nigeria, using an online questionnaire. Data on sociodemographic characteristics, encountered oral and medical conditions, diagnostic practices, and willingness to use saliva diagnostics were collected. Descriptive and inferential statistics were performed using SPSS (version 26), with significance set at *p* < 0.05.

**Results:**

The majority of respondents were aged 20–29 years (52.1%) and had graduated within the past five years (66.4%). While 71.4% reported an awareness of saliva diagnostics, only 23.5% had previously used saliva testing. Dental caries (92.4%), plaque/calculus (73.1%), and tooth wear lesions (42.9%) were the most frequently reported oral conditions, whereas hypertension (93.3%) and diabetes (73.1%) were the most common medical conditions observed among patients. Overall, 64.7% of the respondents expressed a high willingness to adopt saliva-based diagnostics. Willingness was significantly associated with the number of years since graduation (*p* = 0.046) and residency training status (*p* = 0.033), but not with age, gender, or ethnicity.

**Conclusion:**

This pilot study demonstrated a high level of awareness but low utilization of saliva diagnostics, with younger and trainee dentists showing a potentially greater willingness to adopt these tools. Targeted educational initiatives, integration into dental curricula, and supportive policies are required to promote the clinical uptake of saliva-based diagnostics in Nigeria.

## Introduction

Saliva is one of the most essential body fluids and the most important oral fluid, playing key roles in digestion, immunity, buffering, speech, enamel mineralization, protection, and mucosal moisturization [1]. It is a slightly acidic to slightly alkaline (pH of 6.2–7.4) extracellular fluid produced and secreted by three major salivary glands (parotid, submandibular, and sublingual) and numerous (600–1000) salivary glands scattered throughout the oral mucosa [1, 2]. There are two forms of saliva; the serous (watery) and the mucinous (viscous) which in normal function constitutes about 99% water, electrolytes (Na^+^, Ca^2+^, K^+^, Mg^2+^, Cl^−^, HCO_3_^−^, SO_4_^2−^, PO_4_^3−^), mucus, white blood cells, epithelial cells (aids DNA detection), enzymes (ptyalin and lingual lipase), and antimicrobial agents (IgA, lactoferrin and lysozymes) [3].

Saliva is an oral fluid that is increasingly gaining ground among clinicians for monitoring and diagnosing oral and systemic diseases [4]. For centuries, physicians have explored the potential of saliva as a marker of systemic illness [5], however the first clinical validation of saliva testing and approval by the US Food and Drug Administration (FDA) occurred in 1994 [5, 6]. Saliva contains a high amount of Deoxyribonucleic Acid (DNA) and Ribonucleic Acid (RNA) biomarkers, which can aid in the early detection of oral and systemic diseases, including cardiovascular disease, autoimmune and degenerative diseases, and respiratory pathologies [7]. The collection process is non-invasive and convenient for the patient. Skallevold et al. (2021) reported that specific vital biomarkers, including metabolic inflammatory markers, proteomics, genomics, and microbial agents for lung cancer, are now being detected in saliva, which has further helped in the diagnosis and prognosis of the disease [8]. Saliva is the oral diagnostic fluid being used now and will be used the future [9].

Saliva testing has evolved over the years, particularly with the emergence of the coronavirus disease (COVID-19) pandemic, which began in late 2019 [10]. This was also evident in the SARS-CoV-2 testing during the pandemic [11–13]. Although nasopharyngeal (NP) or mid-turbinate (MT) swabs were used with high sensitivity, they were uncomfortable for the subjects being screened; therefore, there was a need for the use of pooled saliva specimens with moderate or high viral load [14]. A study of 162 healthcare workers (100 clinicians and 62 scientists) in Ibadan, Nigeria, found that 80% of clinicians agreed that saliva was the most convenient body fluid to obtain from patients [15]. The majority of respondents were aware of the various functions and importance of saliva as a specimen for clinical and laboratory investigations; however, few had previously used it for such purposes, especially in research [15].

Recently, a non-invasive and rapid screening strategy for SARS-CoV-2 infection in large populations was developed based on a label-free surface-enhanced Raman scattering technique that also utilized machine learning algorithms for the classification of data [16]. Saliva was used as the sample of choice, and the trained machine learning program achieved a prediction accuracy of 95% [16].

Salivary biomarkers can also be used in the diagnosis of dental caries and caries risk assessment using AI (artificial intelligence) models [17]. Koopaie et al. 2021 found that salivary cystatin levels in children with early childhood caries (ECC) were lower than those in caries-free children [18]. In this study, supervised machine learning models were used to assess the usefulness of this biomarker in addition to other patient factors in the prediction of early childhood caries. The use of Cystatin S levels improved the accuracy of other diagnostic methods [18].

There has been increasing interest in the use of saliva and other oral samples for the diagnosis of oral and systemic diseases, and this has drawn significant attention owing to its bio-components that serve as potential biomarkers, ease of collection, cost-effectiveness, and accessible storage [19, 20]. The exploration and isolation of exosomes is an interesting milestone in salivary testing [21]. These exosomes contain certain markers that have also been detected in serum, such as microRNA (miRNA), and further exploration of the exosome in its infant phase has helped improve saliva diagnostic kits [21, 22].

An epidemiological study by Olasehinde et al. (2019) conducted in Ota, Southwestern Nigeria, revealed drug resistance markers for *Plasmodium falciparum* infection with increased diagnostic performance in saliva compared to blood [23]. Furthermore, two-dimensional gel electrophoresis and mass spectrometry have been utilized to identify peptides, resulting in the detection of numerous proteins in a whole saliva specimen [24]. Recent advancements in the incorporation of high-grade liquid chromatography have enabled the detection of saliva peptidomes and proteomes that may be associated with metabolic diseases, such as diabetes mellitus [25].

The possibility of collecting easy and identical information with an oral sample that is non-invasive and readily available may become a preferred alternative to invasive procedures, especially in areas with limited access to healthcare, such as remote/semi-urban geographic locations [26]. Nwoga et al. in 2013 reported the use of Oraquick, an oral fluid (saliva-based) self-test for HIV-1 (Human Immunodeficiency Virus-1) and HIV-2 (Human Immunodeficiency Virus-2) amongst Nigerian patients [27]. The study reported a sensitivity of 98.96% and a specificity of 100% [27]. Despite these positive results, saliva diagnostics is still largely uncommon in Nigeria, and no study has been conducted amongst Nigerian Dentists. The study aimed to assess the level of awareness of dentists in Nigeria regarding saliva-based tests as well as their willingness to utilize salivary diagnostic kits at points of care.

## Methods

### Study design

This descriptive cross-sectional pilot study was conducted in accordance with the STROBE (Strengthening the Reporting of Observational Studies in Epidemiology) guidelines [28].

### Study population

The study population comprised licensed and registered dentists practicing in Nigeria. The data collection was conducted using an online Google Form survey.

### Inclusion and exclusion criteria

We included licensed dentists working in any dental clinic (private and public) in Nigeria who were willing to participate in the survey. Dentists who had retired, were no longer practicing in Nigeria, or who declined participation were excluded.

### Sampling technique and sample size

Convenience sampling was employed in this study. The Kish–Leslie formula was used to determine the minimum required sample size [29], with a prevalence of 0.8 assumed from a previous study [15]. The Kish-Leslie formular is given as n = (Z^2 * p * q) / d^2 [30],

Where:

- n= minimum required sample size
- Z= standard normal deviate (usually 1.96 for 95% confidence level)
- p= estimated prevalence or proportion (from prior studies or pilot study), 0.8 in this case
- q = 1-p
- d= margin of error (precision), taken as 0.05 (5%)

Therefore, a sample size of 246 was estimated

### Data collection procedure

Following ethical approval from the University of Ibadan/University College Hospital (UI/UCH) Ethics Committee (Approval Number: UI/EC/24/0705). The authors contacted potential collaborators for data collection via email advertisements and WhatsApp. Additionally, dentists were sought indirectly through associations such as the Nigerian Dental Association (NDA), the Association of Resident Doctors (ARD), and the Medical and Dental Council of Nigeria (MDCN). Data were collected over a period of four months and nine days (August 29, 2024–January 7, 2025) using a semi-structured, self-administered online questionnaire (Google Forms; Supplementary File 1). Informed consent was obtained electronically before participation. The questionnaire consisted of two sections: (1) sociodemographic information and (2) items assessing the willingness to use saliva test kits.

### Pre-test of the questionnaire

Before the commencement of this study, the adapted questionnaire was tested on the first twenty entries. The feedback obtained from this pre-test was used to refine the instrument, improving its clarity and internal consistency of the items. Responses received from the pre-test of the questionnaire were not used in the final analysis of the data.

### Data analysis method

The completed data were coded and entered into a password-protected computer. The analysis was conducted using the Statistical Package for Social Sciences (SPSS Inc., Chicago, IL, USA, Version 26.0). Descriptive statistics (frequencies, percentages, means, and standard deviations) were used to summarize sociodemographic variables. The willingness to utilize saliva testing was calculated among the respondents by scoring the questions related to willingness in the developed questionnaire. A correct response of “Yes” was scored one, and an incorrect “No” response was scored zero. A 50% benchmark of total obtainable score was then used to classify respondents into having either “High” or “Low” willingness. This binary dichotomization was performed solely for exploratory association analyses. Associations between categorical variables (e.g., gender, specialty, years of practice) and willingness to use saliva test kits were assessed using the chi-squared test (χ²). The level of statistical significance was set at *p* < 0.05.

## Results

### Sociodemographic characteristics of respondents

A total of 119 licensed dentists participated in this study, yielding a response rate of 48.4%. The majority of respondents (52.1%) were within the 20–29 age group, whereas only 3.4% were aged 50–59 years. Males (55.5%) slightly outnumbered females (44.5%). Most participants (66.4%) had graduated within the past 0–5 years, and teaching hospitals (39.5%) were the most common primary place of practice, followed by private ones (32.8%). The Yoruba ethnic group was predominant (79.8%), and most respondents, about three-fourths (75.6%), reported no specialty training (Table 1).

**Table 1:** Demographic characteristics of respondents

Variable	Frequency *N* = 119	Percent 100%
Age		
20–29	62	52.10
30–39	40	33.61
40–49	13	10.92
50–59	4	3.36
Gender at birth		
Female	53	44.54
Male	66	55.46
Number of years since graduation		
0–5 years	79	66.39
6–10 years	16	13.45
11–15 years	14	11.76
16–20 years	5	4.20
> 20 years	5	4.20
Primary place of practice		
Government-owned dental clinic (Non- teaching Hospitals)	28	23.53
Private practice	39	32.77
Teaching hospital	47	39.50
Others	5	4.20
Ethnicity		
Yoruba	95	79.83
Igbo	12	10.08
Others	12	10.08
Specialty training		
In Residency Training	18	15.13
No	89	74.79
Yes	12	10.08

### Oral conditions encountered in dental practice

The most frequently encountered oral conditions were dental caries (92.4%), followed by plaque and calculus (73.1%), and tooth wear lesions (42.9%). Malocclusions (31.1%) and advanced periodontal disease (26.1%) were also common. In contrast, rare conditions, such as temporomandibular joint (TMJ) disorders (2.5%), congenital anomalies (1.7%), and oral cancer (5.0%) have been infrequently reported. About 1 in 5 of the respondents (20.17%) reported encountering advanced sequelae of dental caries such as necrotizing fasciitis (Table 2).

**Table 2:** Most Common Oral Conditions Seen by Dental Practitioners

Variable	Frequency *N* = 119	Percent 100%
Most common conditions		
In your practice, what are the most common oral conditions you see in your patients?		
Carious lesions/Cavities		
No	9	7.56
Yes	110	92.44
Advanced Sequalae of Dental Caries e.g. Submandibular abscess/Necrotizing Fasciitis		
No	95	79.83
Yes	24	20.17
Halitosis		
No	94	78.99
Yes	25	21.01
Plaque and Calculus		
No	32	26.89
Yes	87	73.11
Advanced gingiva/Periodontal Diseases		
No	88	73.95
Yes	31	26.05
Tooth discolorations		
No	102	85.71
Yes	17	14.29
Tooth wear lesions		
No	68	57.14
Yes	51	42.86
Trauma/Dentofacial injuries		
No	100	84.03
Yes	19	15.97
TMJ Conditions		
No	116	97.48
Yes	3	2.52
Dentofacial Neoplasms/Oral Cancer		
No	113	94.96
Yes	6	5.04
Congenital Anomalies		
No	117	98.32
Yes	2	1.68
Malocclusions		
No	82	68.91
Yes	37	31.09
Oral medicine conditions e.g. autoimmune disorders or vitamin deficiencies		
No	117	98.32
Yes	2	1.68
Oral infections e.g. candidiasis, herpes		
No	118	99.16
Yes	1	0.84

### Medical conditions commonly seen in dental patients

Hypertension (93.3%) and diabetes (73.1%) were the most frequently reported systemic medical conditions. Peptic or gastric ulcers were also common, affecting 68.1% of cases. Asthma (8.4%), congenital disorders (5.0%), and autoimmune diseases (0.8%) were less commonly encountered (Table 3).

**Table 3:** Most common medical conditions encountered in dental practice

Variable	Frequency *N* = 119	Percent 100%
In your practice, what are the most common medical conditions you see in your patients?		
Hypertension		
No	8	6.72
Yes	111	93.28
Diabetes		
No	32	26.89
Yes	87	73.11
Autoimmune conditions		
No	118	99.16
Yes	1	0.84
Congenital conditions		
No	113	94.96
Yes	6	5.04
Asthma		
No	109	91.60
Yes	10	8.40
Peptic/Gastric Ulcer		
No	38	31.93
Yes	81	68.07

### Routine diagnostic practices and awareness of saliva diagnostics

More than half of the respondents (57.1%) did not routinely order ancillary tests. Among those, blood tests were the most frequently requested (74.8%), followed by urine tests (5.0%). Awareness of saliva diagnostics was relatively high, with 71.4% of the respondents reporting familiarity. Furthermore, 77.3% indicated that they were aware of the conditions detectable with saliva, 10.9% were uncertain, and 8.4% reported no awareness. Despite this, only a minority used saliva as a diagnostic medium (Table 4).

**Table 4:** Diagnostic practices and awareness of saliva-based testing (*n* = 119)

Variable	Category	*n*	%
Routinely order ancillary tests	No	68	57.10
	Yes	51	42.90
Type of diagnostic/screening tests*	Blood test	89	74.80
	Urine test	6	5.00
	Blood pressure measurement	3	2.50
	Other diagnostic tests†	3	2.50
Awareness of saliva diagnostics	Yes	85	71.40
	No	21	17.60
	Uncertain	13	10.90
Aware of conditions detectable with saliva	Yes	92	77.30
	Not sure	17	14.30
Willingness		10	8.40
High	**75(63.03)**		
Low	**44(36.97)**		

*Respondents could select multiple diagnostic tests and the question was not required

†Other includes biopsy, OPG, random blood sugar (RBS); reported by < 3% of respondents

**Fig. 1:**
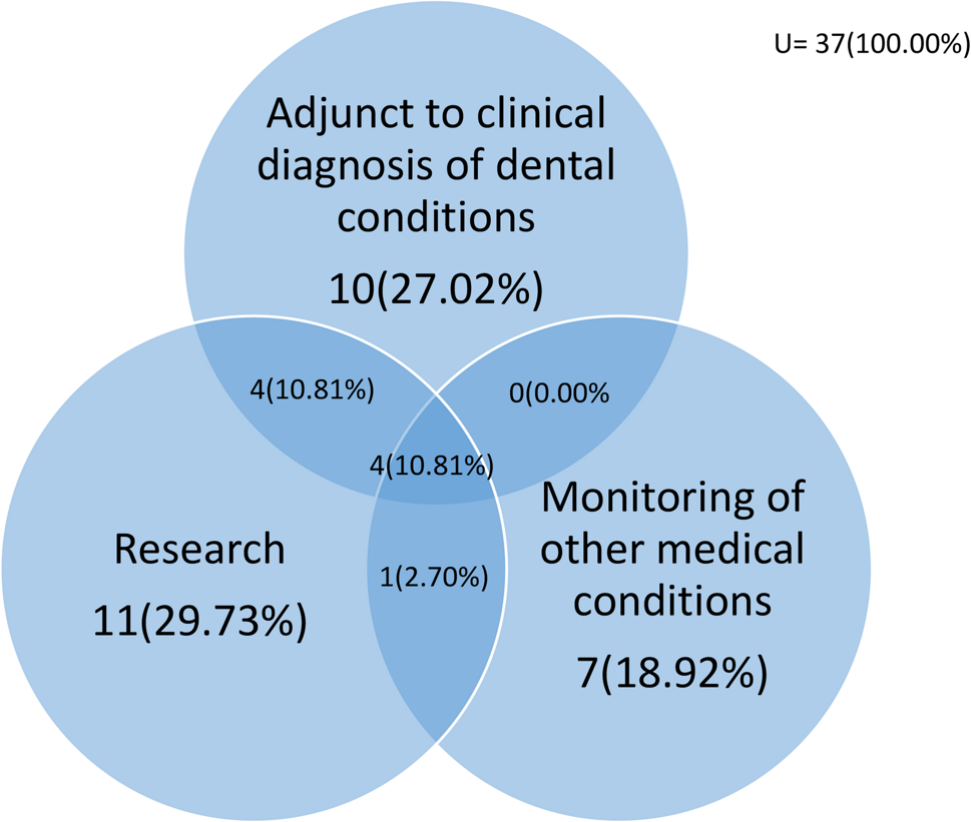
A Venn diagram showing the distribution of respondents to the purpose of saliva testing

Figure 1 shows that of the 37 (31.1%) respondents that have utilized saliva testing, more than half (29.7% and 27.0%) does so for “Research” and “Adjunct to clinical diagnosis of dental conditions” respectively, a fraction (18.9%) utilizes the test for monitoring of other medical conditions and only about a tenth (10.8%) utilizes the test for all three purposes.

### Association between respondents’ characteristics and willingness to use saliva diagnostics

The majority of dentists (64.7%) expressed a high willingness to use salivary diagnostics in their practice. Willingness was significantly associated with the number of years since graduation and completion of residency training. Dentists who had graduated within the past 10 years were more likely to express willingness (*p* = 0.046), and those currently undergoing residency training also demonstrated higher acceptance (*p* = 0.033). However, no significant associations were observed with age, sex, ethnicity, or place of practice (Table 5).

**Table 5:** Association Between Respondents' Characteristics and Willingness to Use Saliva Diagnostics

Variable	Willingness			chi2	*p*
	High *n*(%)	Low *n*(%)	Total		
Age					
20–29	36(58.06)	26(41.94)	62		
30–39	29(72.50)	11(27.50)	40		0.404*f*
40–49	7(53.85)	6(46.15)	13		
50–59	3(75.00)	1(25.00)	4		
Gender					
Female	31(58.49)	22(41.51)	53	0.8432	0.358
Male	44(66.67)	22(33.33)	66		
Year after graduation					
0–5 years	46(58.23)	33(41.77)	79		
6–10 years	14(87.50)	2(12.50)	16		0.046*f*
11–15 years	8(57.14)	6(42.86)	14		
16–20 years	2(40.00)	3(60.00)	5		
> 20 years	5(100.00)	0(0.00)	5		
Place of practice					
Government-owned dental clinic (Non- teaching Hospitals)	16(57.14)	12(42.86)	28		
Private practice	24(61.54)	15(38.46)	39		0.796*f*
Teaching hospital	31(65.96)	16(34.04)	47		
Others	4(80.00)	1(20.00)	5		
Ethnicity					
Yoruba	61(64.21)	34(35.79)	95		
Igbo	8(66.67)	4(33.33)	12		0.615*f*
Others	6(50.00)	6(50.00)	12		
Specialty in training					
In Residency Training	16(88.89)	2(11.11)	18		
No	51(57.30)	38(42.7)	89		0.033*f*
Yes	8(66.67)	4(33.33)	12		

*f* = Fischer exact test

* Values are presented as numbers (percentage). Percentages are presented as row percentages. Chi-square tests were used, and Fisher’s exact test was applied when the expected cell counts were < 5. *p* < 0.05 considered statistically significant

## Discussion

This pilot study gives a preliminary assessment of the disposition of Nigerian dentists to saliva diagnostics. Saliva remains one of the most cost-effective and straightforward diagnostic tool for detecting oral diseases. The use of saliva instead of other body fluids (blood, serum, cerebrospinal fluid, and lymph) can be attributed to their ease of collection, reduced risk of infection, and reduced cost, a triple health aim approach [31, 32]. These advantages make dentists more willing to use saliva as a diagnostic tool, especially in rural areas where high-tech infrastructure is not available [33]. This trend is consistent with Greenberg et al. (2010), who reported that dentists were more inclined to use saliva as a diagnostic tool compared to blood samples obtained by finger prick or other body fluids [34].

Our study findings revealed that most participants (71.4%) were aware of saliva diagnostics, and 28.6% had little or no knowledge. Despite this awareness, only 23.5% had utilized saliva testing in any form, while 74.8% reported not using it. This finding aligns with a previous work by Lasisi et al., who reported that 95.7% of Nigerian healthcare workers were aware that saliva could be used for clinical and laboratory tests [15]. Respondents highlighted that saliva is a valuable tool in clinical tests and disease diagnosis due to its ability to provide real-time results [15]. However, our study specifically focused on dentists, revealing slightly lower, but still substantial, awareness levels.

While 40% of respondents believed there were no significant challenges in implementing saliva-based diagnostics, 37.5% identified potential barriers, including cost concerns, patient skepticism, and technical difficulties such as saliva flow variability and reagent availability. Research on saliva diagnostics implementation in resource-limited settings has consistently identified similar obstacles, including a lack of facilities, inadequate funding for research activities, and limited correlation between plasma and saliva levels for specific parameters [35]. The cost barrier is particularly relevant in Nigeria, where healthcare spending constraints significantly impact the adoption of new technologies [36]. When dentists were asked about their willingness to incorporate saliva testing into their practice, 64.7% expressed openness to using it for diagnosing medical or dental conditions. However, 18.5% remained uncertain, while 16.8% were opposed. These findings suggest a general receptiveness to saliva diagnostics despite potential systemic challenges.

This study also revealed that dentists routinely screened patients for other medical conditions and provided data on the most common forms of medical conditions seen at their practices. The clinical conditions most frequently encountered by our respondents include; carious lesions (92.4%), plaque and calculus (73.1%), and periodontal diseases — may not be ideal candidates for routine applications of salivary diagnostics. The high prevalence of hypertension (93.3%) and diabetes (73.1%) among patients seen by our respondents may indicate potential for saliva-based screening of systemic conditions, as these conditions have salivary biomarkers [32, 37–39].

Our findings are consistent with those of Greenberg et al. on dentists’ attitudes towards chairside screening of medical conditions [40]. In Greenberg’s 2010 study conducted among USA (United States of America) dentists, 87.7% of the 1,945 respondents were willing to collect oral fluids for salivary diagnostic tests [34], which is higher than our report of 71.43%. This may be due to protocols/norms/patterns of collecting body fluids for diagnosis amongst Nigerian trained dentists. Furthermore, dentists with less than 10 years of clinical experience and those in residency training were more willing to utilize salivary diagnostic tests. This suggests that recent graduate dentists and those undergoing academic/clinical training may be more open to trying new diagnostic kits.

Results from the study by Rathnayake et al., a sample of 1000 Swedish adults in 2013, found that IL-8 (Interleukin 8) concentration was 2 times higher in people with neoplastic conditions and that MMP-8 (Matrix Metalloprotease 8) was higher in patients with diabetes, cardiovascular conditions, and muscle/joint disorders and these differences were found to be significant even after adjusting for age, gender and smoking habits [41]. Furthermore, recent advances in saliva test kits, incorporating emerging technologies such as spectroscopy, lab-on-a chip sensor, hormones, multiplexing and artificial intelligence/machine learning, in real time detection, diagnosis, prognosis and prediction of oral and systemic diseases [37, 38, 42–44].

## Limitations

This study has provided great insight into this topic; there were however several limitations while conducting this study. The total number of participants in this study (119) was lower than the calculated sample size (246), reducing the statistical power of the study, and increasing the risk of type II error. Additionally, the sample was not well-representative (convenience sampling) of the population studied (selection bias), as evidenced by the 79.8% representation of the Yoruba ethnic group. Notably, some respondents expressed a limited understanding of salivary diagnostics, reflecting the perspectives of a predominantly early-career cohort rather than the entire Nigerian dental workforce, which may have also influenced their responses. This highlights a need for education and awareness about this topic. Future studies should use a validated willingness scale or full Likert-scale to assess willingness to use salivary diagnostics. Particularly, our study did not evaluate the feasibility of integrating this into current dental practices in Nigerian dental clinics. Another possible limitation is the recall bias that may have occurred while filling out the online self-reported questionnaire, which may have affected the results.

## Recommendations

Future studies can focus on expanding the sample size and improving the representativeness of the sample via potential randomization. The barriers that were identified (cost, procedural issues, and technical difficulties) can be further explored and investigated to evaluate the feasibility of adoption including consumer perceptions [45]. To promote the adoption of saliva-based diagnostics among dentists in Nigeria, it is essential to include saliva diagnostics in the training curriculum for dental professionals and to implement more advocacy and supportive policies that help overcome the barriers to adoption.

Incorporating machine learning into saliva diagnostics can be a game changer for pathologists [46]. Enhanced algorithmic models have shown great potential for pattern recognition in diagnosis and predictions [46, 47]. Finally, in an era of constant innovation, we encourage more local research using saliva diagnostics to enhance African precision in oral healthcare [48]. There are gaps and opportunities to generate more novel insights from DNA/RNA extraction of saliva samples from the Nigerian population, which can be integral to outbreak preparedness and ultimately strengthen National biosecurity.

## Conclusion

The pilot study provides valuable insights into the current awareness and willingness among Nigerian dentists to adopt ancillary salivary diagnostic kits. While there may be challenges and barriers to utilizing saliva test kits, such as costs, technical difficulties, and procedural issues, there is a need for educational programs and policy-making efforts to enhance the utilization of saliva-based diagnostics in dental practices across Nigeria. Generally, the findings from this study provide positive insights into the willingness of dentists to utilise salivary diagnostic tests. Future studies should expand the sample size and geographic representation, and beyond assessing willingness, also examine the clinical knowledge, diagnostic accuracy, and procedural confidence of dentists in using salivary kits.

## Data Availability

The datasets generated and/or analyzed during the current study are available from the corresponding author upon reasonable request.

## Abbreviations

AI: Artificial Intelligence
ANOVA: Analysis of Variance
COVID: 19–Coronavirus Disease 2019
FDA: Food and Drug Administration
miRNA: MicroRNA
Na^+^: Sodium Ion
Ca^2+^: Calcium Ion
K^+^: Potassium Ion
Mg^2+^: Magnesium Ion
Cl^−^: Chlorine Ion
HCO₃^−^: Bicarbonate Ion
SO_4_^2−^: Sulphate Ion
PO_4_^3−^: Phosphate Ion
MT: Mid–Turbinate
NP: Nasopharyngeal
DNA: Deoxynucleic Acid
RNA: Ribonucleic Acid
SARS: CoV–2–Severe Acute Respiratory Syndrome Coronavirus 2
HIV: Human Immunodeficiency Virus
SPSS: Statistical Package for the Social Sciences
STROBE: Strengthening the Reporting of Observational Studies in Epidemiology
TMJ: Temporomandibular Joint
UCH: University College Hospital
UI: University of Ibadan
IL: Interleukin
MMP: Matrix Metalloprotease
USA: United States of America

## References

[1] Humphrey SP, Williamson RT. A review of saliva: Normal composition, flow, and function. J Prosthet Dent. 2001;85:162–9. 10.1067/mpr.2001.113778. 11208206 10.1067/mpr.2001.113778

[2] Dodds M, Roland S, Edgar M, Thornhill M. Saliva A review of its role in maintaining oral health and preventing dental disease. BDJ. 2015;2:15123. 10.1038/bdjteam.2015.123.

[3] Tiwari M. Science behind human saliva. J Nat Sci Biol Med. 2011;2:53. 10.4103/0976-9668.82322. 22470235 10.4103/0976-9668.82322 PMC3312700

[4] Albagieh H, Alshehri AZ, Alduraywishi AS, Aldaws A, AlBalawi SS, Abu Shaqqaf HF, et al. Evaluation of Salivary Diagnostics: Applications, Benefits, Challenges, and Future Prospects in Dental and Systemic Disease Detection. Cureus. 2025;17. 10.7759/CUREUS.77520. 10.7759/cureus.77520 PMC11830415 39958008

[5] Ogbureke KUE, Ogbureke EI. The history of salivary diagnostics. Advances in Salivary Diagnostics. Berlin Heidelberg: Springer; 2015. pp. 17–31. 10.1007/978-3-662-45399-5_2.

[6] Seachrist L, FDA approves saliva AIDS test - UPI Archives. 1994 https://www.upi.com/Archives/1994/12/23/FDA-approves-saliva-AIDS-test/3018788158800/. Accessed 7 Dec 2025.

[7] Kumari S, Samara M, Ampadi Ramachandran R, Gosh S, George H, Wang R et al. A Review on Saliva-Based Health Diagnostics: Biomarker Selection and Future Directions. Biomedical materials & devices (New York, NY). Biomed Mater Devices; 2023;2:121–38. 10.1007/S44174-023-00090-Z. 10.1007/s44174-023-00090-z PMC10243891 37363139

[8] Skallevold HE, Vallenari EM, Sapkota D. Salivary Biomarkers in Lung Cancer. Mediators Inflamm. Mediators Inflamm; 2021;2021. 10.1155/2021/6019791. 10.1155/2021/6019791 PMC8528626 34690552

[9] Albagieh H, Alshehri AZ, Alduraywishi AS, Aldaws A, AlBalawi SS, Abu Shaqqaf HF, et al. Evaluation of Salivary Diagnostics: Applications, Benefits, Challenges, and Future Prospects in Dental and Systemic Disease Detection. Cureus. Springer Science and Business Media LLC; 2025. 10.7759/cureus.77520. 10.7759/cureus.77520 PMC11830415 39958008

[10] de Oliveira Neto NF, Caixeta RAV, Zerbinati RM, Zarpellon AC, Caetano MW, Pallos D, et al. The Emergence of Saliva as a Diagnostic and Prognostic Tool for Viral Infections. Viruses. 2024;16. 10.3390/V16111759. 10.3390/v16111759 PMC11599014 39599873

[11] Fiorani-Nascimento R, Marques KL, de Carvalho VJ, Dias FP, Cabral JM, dos Santos CWN, et al. Accuracy of saliva tests for SARS-CoV-2 diagnosis during the pandemic in Rio de Janeiro, Brazil. Sci Rep. 2025;15:36932. 10.1038/s41598-025-20841-w. 41125663 10.1038/s41598-025-20841-w PMC12546890

[12] Sultana N, Biswas PP, Resma TI, Fatema N, Sharmin R, Sweety AA, et al. Evaluating saliva for SARS-CoV-2 detection: A practical alternative. Diagn Microbiol Infect Dis. 2025;113:116902. 10.1016/j.diagmicrobio.2025.116902. 40398133 10.1016/j.diagmicrobio.2025.116902

[13] Tan SH, Allicock OM, Katamba A, Carrington CVF, Wyllie AL, Armstrong-Hough M. Saliva-based methods for SARS-CoV-2 testing in low-and middle-income countries. Bull World Health Organ World Health Organ. 2022;100:808–14. 10.2471/BLT.22.288526. 10.2471/BLT.22.288526 PMC9706358 36466209

[14] Pinninti S, Trieu C, Pati SK, Latting M, Cooper J, Seleme MC, et al. Comparing Nasopharyngeal and Midturbinate Nasal Swab Testing for the Identification of Severe Acute Respiratory Syndrome Coronavirus 2. Clin Infect Dis. 2021;72:1253–5. 10.1093/CID/CIAA882. 32596725 10.1093/cid/ciaa882 PMC7337631

[15] Lasisi TJ, Lawal FB. Preference of saliva over other body fluids as samples for clinical and laboratory investigations among healthcare workers in Ibadan, Nigeria. Pan Afr Med J. 2019;34:191. 10.11604/PAMJ.2019.34.191.18738. 32180865 10.11604/pamj.2019.34.191.18738 PMC7060920

[16] Qin J, Tian X, Liu S, Yang Z, Shi D, Xu S, et al. Rapid classification of SARS-CoV-2 variant strains using machine learning-based label-free SERS strategy. Talanta. 2024;267. 10.1016/j.talanta.2023.125080. 10.1016/j.talanta.2023.125080 37678002

[17] Constantin V, Luchian I, Goriuc A, Budala DG, Bida FC, Cojocaru C, et al. Salivary Biomarkers Identification: Advances in Standard and Emerging Technologies. Oral. 2025;5:26. 10.3390/ORAL5020026.

[18] Koopaie M, Salamati M, Montazeri R, Davoudi M, Kolahdooz S. Salivary cystatin S levels in children with early childhood caries in comparison with caries-free children; statistical analysis and machine learning. BMC Oral Health. 2021;21. 10.1186/S12903-021-02016-X. 10.1186/s12903-021-02016-x PMC8683819 34922509

[19] Khurshid Z, Zafar MS, Khan RS, Najeeb S, Slowey PD, Rehman IU. Role of Salivary Biomarkers in Oral Cancer Detection. Adv Clin Chem. 2018;86:23–70. 10.1016/bs.acc.2018.05.002. 30144841 10.1016/bs.acc.2018.05.002

[20] Öztürk VÖ, Emingil G, Umeizudike K, Tervahartiala T, Gieselmann DR, Maier K, et al. Evaluation of active matrix metalloproteinase-8 (aMMP-8) chair-side test as a diagnostic biomarker in the staging of periodontal diseases. Arch Oral Biol. 2021;124. 10.1016/j.archoralbio.2020.104955. 10.1016/j.archoralbio.2020.104955 33556789

[21] Dilsiz N. A comprehensive review on recent advances in exosome isolation and characterization: Toward clinical applications. Transl Oncol. 2024;50. 10.1016/j.tranon.2024.102121. 10.1016/j.tranon.2024.102121 PMC11418158 39278189

[22] Mukerjee N, Bhattacharya A, Maitra S, Kaur M, Ganesan S, Mishra S, et al. Exosome isolation and characterization for advanced diagnostic and therapeutic applications. Mater Today Bio. 2025;31. 10.1016/j.mtbio.2025.101613. 10.1016/j.mtbio.2025.101613 PMC11950786 40161926

[23] Olasehinde GI, Diji-Geske RI, Fadina I, Arogundade D, Darby P, Adeleke A, et al. Epidemiology of Plasmodium falciparum infection and drug resistance markers in Ota Area, Southwestern Nigeria. Infect Drug Resist. 2019;12:1941–9. 10.2147/IDR.S190386. 31308714 10.2147/IDR.S190386 PMC6616117

[24] Hu S, Jiang J, Wong DT. Proteomic Analysis of Saliva: 2D Gel Electrophoresis, LC-MS/MS, and Western Blotting. Methods Mol Biol. 2010;666:31. 10.1007/978-1-60761-820-1_3. 20717776 10.1007/978-1-60761-820-1_3 PMC3209956

[25] Chen S, Lai W, Wang H. Recent advances in high-performance liquid chromatography tandem mass spectrometry techniques for analysis of DNA damage and epigenetic modifications. Mutat Res Genet Toxicol Environ Mutagen. 2024;896. 10.1016/j.mrgentox.2024.503755. 10.1016/j.mrgentox.2024.503755 38821674

[26] Valinetz ED, Cangelosi GA. A Look Inside: Oral Sampling for Detection of Non-oral Infectious Diseases. J Clin Microbiol. 2021;59. 10.1128/JCM.02360-20. 10.1128/JCM.02360-20 PMC8451410 33888590

[27] Nwoga MC, Odukoya O, Savage KO, Effiom OA. Evaluation of accuracy of OraQuick^®^ rapid test in detecting HIV antibodies in saliva of Nigerians. Niger Dent J. 2012;20:66–9. 10.61172/NDJ.V20I2.108.

[28] Vandenbroucke JP, Von Elm E, Altman DG, Gøtzsche PC, Mulrow CD, Pocock SJ, et al. Strengthening the Reporting of Observational Studies in Epidemiology (STROBE): explanation and elaboration. Epidemiology. 2007;18:805–35. 10.1097/EDE.0B013E3181577511. 18049195 10.1097/EDE.0b013e3181577511

[29] Wiegand H, Kish L: Survey Sampling. John Wiley, Sons I, York N, IX + 643 S. London, 31 Abb., 56 Tab., Preis 83 s. Biom Z. John Wiley & Sons, Ltd; 1968;10:88–9. 10.1002/BIMJ.19680100122.

[30] Kish L. Survey sampling | WorldCat.org. 1965. (Accessed 7 Dec, 2025). https://search.worldcat.org/title/survey-sampling/oclc/256017

[31] Kokko P. Improving the value of healthcare systems using the Triple Aim framework: A systematic literature review. Health Policy (New York). 2022;126:302–9. 10.1016/J.HEALTHPOL.2022.02.005. 10.1016/j.healthpol.2022.02.005 35221120

[32] Anwar M, Alam BF, Ali S, Tariq SF, Aali K, Abrar E, et al. Evaluation of Salivary Mucin, Amylase, Protein Profile, and Periodontal Parameters among Hypertensive and Diabetic Patients. Appl Sci. 2022;12:7407. 10.3390/APP12157407.

[33] Bouhairi EL, Lachheb O, Sidqui M. Exploring the Efficacy and Applications of Salivary Tests in Modern Dental Practice. 2024; 10.5281/zenodo.10529724.

[34] Greenberg BL, Glick M, Julie FH, Kantor ML. Dentists’ attitudes toward chairside screening for medical conditions. J Am Dent Assoc. 2010;141:52–62. 10.14219/jada.archive.2010.0021. 20045822 10.14219/jada.archive.2010.0021

[35] Li Y, Ou Y, Fan K, Liu G. Salivary diagnostics: opportunities and challenges. Theranostics Theranostics. 2024;14:6969–90. 10.7150/THNO.100600. 39629130 10.7150/thno.100600 PMC11610148

[36] Awoyemi BO, Makanju AA, Mpapalika J, Ekpeyo RS. A time series analysis of government expenditure and health outcomes in Nigeria. J Public Health Afr. 2023;14. 10.4081/JPHIA.2023.1409. 10.4081/jphia.2023.1409 PMC10481895 37680869

[37] Miller CS, Foley JD, Floriano PN, Christodoulides N, Ebersole JL, Campbell CL, et al. Utility of Salivary Biomarkers for Demonstrating Acute Myocardial Infarction. J Dent Res. 2014;93:72S. 10.1177/0022034514537522. 24879575 10.1177/0022034514537522 PMC4107546

[38] González V, Martró E, Folch C, Esteve A, Matas L, Montoliu A, et al. Detection of hepatitis C virus antibodies in oral fluid specimens for prevalence studies. Eur J Clin Microbiol Infect Dis. 2008;27:121–6. 10.1007/S10096-007-0408-Z. 18027006 10.1007/s10096-007-0408-z

[39] Cenzato N, Cazzaniga F, Maspero C, Tartaglia GM, Del Fabbro M. SALIVA-based diagnostic approach for diabetes mellitus: a step towards non-invasive detection - a scoping review. Eur Rev Med Pharmacol Sci. 2023;27:12080–7. 10.26355/EURREV_202312_34806. 38164870 10.26355/eurrev_202312_34806

[40] Greenberg BL, Glick M, Frantsve-Hawley J, Kantor ML. Dentists’ attitudes toward chairside screening for medical conditions. J Am Dent Assoc Engl. 2010;141:52–62. 10.14219/jada.archive.2010.0021. 10.14219/jada.archive.2010.0021 20045822

[41] Rathnayake N, Åkerman S, Klinge B, Lundegren N, Jansson H, Tryselius Y, et al. Salivary biomarkers for detection of systemic diseases. PLoS ONE. 2013;8. 10.1371/JOURNAL.PONE.0061356. 10.1371/journal.pone.0061356 PMC3634781 23637817

[42] Han GR, Goncharov A, Eryilmaz M, Ye S, Palanisamy B, Ghosh R, et al. Machine learning in point-of-care testing: innovations, challenges, and opportunities. Nat Commun. 2025;16:3165. 10.1038/s41467-025-58527-6. 40175414 10.1038/s41467-025-58527-6 PMC11965387

[43] Paluszkiewicz C, Pięta E, Woźniak M, Piergies N, Koniewska A, Ścierski W, et al. Saliva as a first-line diagnostic tool: A spectral challenge for identification of cancer biomarkers. J Mol Liq. 2020;307:112961. 10.1016/J.MOLLIQ.2020.112961.

[44] Song M, Bai H, Zhang P, Zhou X, Ying B. Promising applications of human-derived saliva biomarker testing in clinical diagnostics. Int J Oral Sci. 2023;15. 10.1038/S41368-022-00209-W. 10.1038/s41368-022-00209-w PMC9810734 36596771

[45] Sun R, Amidi S, Yee JY, Liaw A, Hosseinpour S, Ivanovski S, et al. A survey of Australian consumers’ and dentists’ perceptions of saliva as a biofluid for periodontitis diagnosis. BMC Oral Health. 2025;25. 10.1186/s12903-025-07062-3. 10.1186/s12903-025-07062-3 PMC12553151 41139198

[46] Patil S, Albogami S, Hosmani J, Mujoo S, Kamil MA, Mansour MA, et al. Artificial Intelligence in the Diagnosis of Oral Diseases: Applications and Pitfalls. Diagnostics. 2022;12:1029. 10.3390/DIAGNOSTICS12051029. 35626185 10.3390/diagnostics12051029 PMC9139975

[47] Kokkotis C, Moustakidis S, Swift SJ, Kontopidou F, Kavouras I, Doulamis A, et al. Artificial Intelligence and Machine Learning in the Diagnosis and Prognosis of Diseases Through Breath Analysis: A Scoping Review. Information. 2025;16:968. 10.3390/INFO16110968.

[48] Alufa O, Tundealao S, Adisa A. The Use of Advanced Oral Cancer Diagnostic Tools in Africa: Potential and Challenges. J Oral Health Comm Dent. 2025. 10.5005/jp-journals-10062-0203.

